# Comparison of Electropolishing of Aluminum in a Deep Eutectic Medium and Acidic Electrolyte

**DOI:** 10.3390/molecules25235712

**Published:** 2020-12-03

**Authors:** Tarek M. Abdel-Fattah, J. Derek Loftis

**Affiliations:** 1 Applied Research Center at Thomas Jefferson National Accelerator Facility and Department of Molecular Biology and Chemistry at Christopher Newport University, Newport News, VA 23606, USA; derek.loftis.04@cnu.edu; 2 Department of Physical Sciences, Virginia Institute of Marine Science, College of William & Mary, Williamsburg, VA 23187, USA

**Keywords:** ionic liquid, electrochemical polishing, choline chloride, phosphoric acid, surface characterization, atomic force microscopy

## Abstract

Research advances in electropolishing, with respect to the field of metalworking, have afforded significant improvements in the surface roughness and conductivity properties of aluminum polished surfaces in ways that machine polishing and simple chemical polishing cannot. The effects of a deep eutectic medium as an acid-free electrolyte were tested to determine the potential energy thresholds during electropolishing treatments based upon temperature, experiment duration, current, and voltage. Using voltammetry and chronoamperometry tests during electropolishing to supplement representative recordings via atomic force microscopy (AFM), surface morphology comparisons were performed regarding the electropolishing efficiency of phosphoric acid and acid-free ionic liquid treatments for aluminum. This eco-friendly solution produced polished surfaces superior to those surfaces treated with industry standard acid electrochemistry treatments of 1 M phosphoric acid. The roughness average of the as-received sample became 6.11 times smoother, improving from 159 nm to 26 nm when electropolished with the deep eutectic solvent. This result was accompanied by a mass loss of 0.039 g and a 7.2 µm change in step height along the edge of the electropolishing interface, whereas the acid treatment resulted in a slight improvement in surface roughness, becoming 1.63 times smoother with an average post-electropolishing roughness of 97.7 nm, yielding a mass loss of 0.0458 g and a step height of 8.1 µm.

## 1. Introduction

Electrochemical polishing (electropolishing) is the controlled corrosion of metal surfaces [1,2,3]. The concept behind this mechanism of corroding metals with liquids is to yield a reduction in the surface roughness of the polished metals [4,5]. Another major benefit of electropolishing over surface buffing alternatives is the practical application of reducing surface roughness and impurities to nearly negligible quantities on polished surfaces [6,7]. Currently, large quantities of surface-polished products are being treated with hazardous chemical solutions [8,9,10]. Phosphoric and sulfuric acid mixtures account for a plurality of these acid electropolishing treatments in pure metals and alloys [11,12].

The benefits of electrochemical polishing have gained notoriety, being determined to be an ideal method for improving a metal’s optimum roughness while also greatly improving electrical conductivity [6,7,12]. Many acid treatments currently utilized for electropolishing metal surfaces provide an ideal mirror finish by removing the exposed surface layer of the sheet metal. However, acid solutions provide this clean electropolished finish to the metal at the expense of hydrogen contamination [4,9,12]. The removal of hydrogen contamination generally entails the use of high-temperature treatments in excess of 800 °C for several hours in vacuo [13]. 

High-purity metal samples of aluminum were tested to determine the effectiveness of an industry standard acid solution of 1 M phosphoric acid and an ionic liquid medium composed of ethylene glycol and choline chloride mixed in a 2:1 molar ratio. Previous studies demonstrated the effectiveness of various conformities of this ionic liquid with stainless steel and other alloys [1,5,10]. It was the aim of this investigation to optimize the electrochemistry tests to produce a superior polish of this ionic liquid and to weigh the benefits of this ionic liquid against an industry standard 1 M phosphoric acid electropolishing treatment. 

## 2. Results

**Figure 1:**
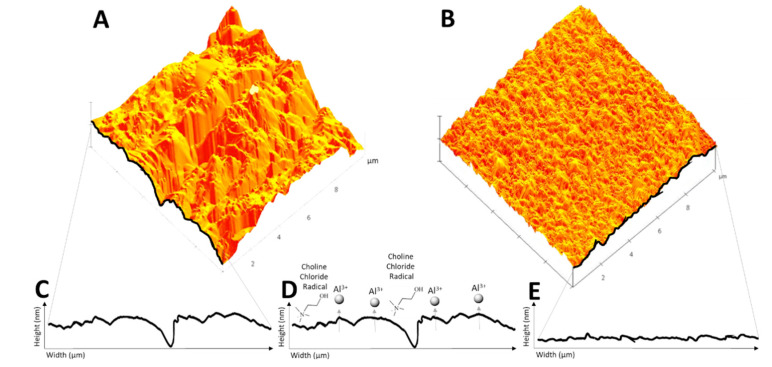
Schematic conceptualization of anodic leveling of aluminum surfaces via atomic force microscopy (AFM) at different phases of the study: (**A**) before electropolishing, and (**B**) after electropolishing treatments with the ionic liquid, with representative 2D surface profiles depicted (**C**) before, (**D**) during, and (**E**) after experiments.

**Figure 2:**
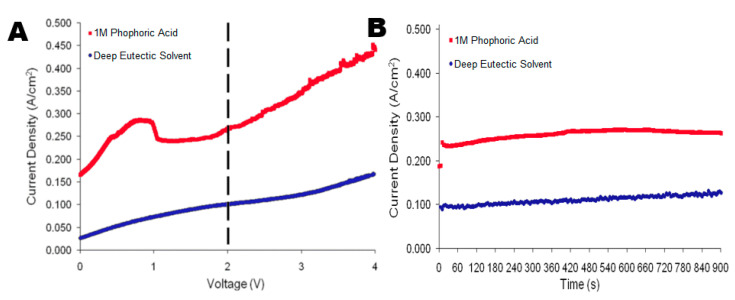
Linear sweep voltammograms (**A**) for both polishing agents with aluminum samples stepped from 0 to 4 V at a constant scan rate of 20 mVs^−1^. The dashed line indicates the ideal voltage utilized for chronoamperometry. (**B**) Chronoamperometry for phosphoric acid (3.51 A/cm^2^) and the ionic liquid eutectic (1.08 A/cm^2^) with high-purity aluminum samples.

The general mechanism of the electropolishing of metal surfaces in this study using the ionic liquid deep eutectic solvent electrolyte is represented in Figure 1. The optimum conditions for the steady electropolishing of aluminum metals with the ionic liquid were revealed to be 2 V for 900 s via linear sweep voltammetry, as shown in Figure 2. Set to progress at a scan rate of 20 mVs^−1^ from 0 to 8 V, the effects of electropolishing became erratic and irreproducible beyond 4 V. A point of inflection present at 0.098 A/cm^2^ in the linear sweep voltammogram indicated the optimum conditions for chronoamperometry for ionic liquid treatments in aluminum samples with a current range of 0.092 to 0.11 A/cm^2^ (Figure 2A). Chronoamperograms of the high-purity aluminum samples were run during the polishing process using phosphoric acid at an average current of 3.51 A/cm^2^ and at 1.08 A/cm^2^ with the ionic liquid eutectic (Figure 2B). Mass comparison of the original weight of aluminum prior to electropolishing reported a difference of 0.039 g when compared with the post-polishing weight of 5.79 g for aluminum polished via the ionic liquid method (Table 1). 

**Figure 3:**
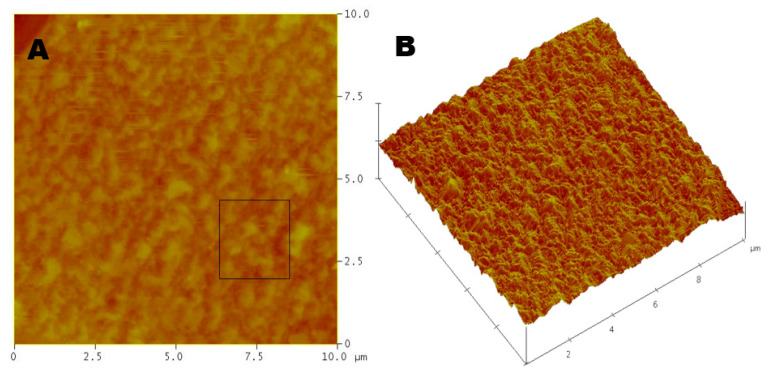
Atomic force microscopy of aluminum post-electropolishing with the ionic liquid in both (**A**) two and (**B**) three dimensions—recording an average roughness of 26 ± 2 nm by utilizing the root mean square method for calculation. A 10 × 10 µm recording region was utilized.

Prior to electropolishing, the aluminum measured a root mean square roughness average of 159 ± 9 nm, while after polishing treatments with the ethylene glycol and choline chloride solution yielded a roughness of 26 ± 2 nm via atomic force microscopy (AFM) (Table 2, Figure 3). The relative smoothing efficiency with the ionic liquid revealed a roughness difference of 132 nm for aluminum acid-free polishing for an overall electropolishing efficiency of 83.2% (Table 2). Step height measurements disclosed the division between the polished and unpolished regions to be comparable with a weight difference of 7.2 µm (Table 3).

Phosphoric acid polishing treatments for the aluminum samples demonstrated a peak value of 0.286 A/cm^2^ at 1 V with a local minimum of 0.23 A/cm^2^ immediately following at 1.5 V according to the linear sweep voltammetry analysis (Figure 2A). This yielded a current range of 0.23 to 0.25 A/cm^2^ for chronoamperometry (Figure 2B) as the linear sweep progressed from 0 to 8 V at a scan rate of 20 mVs^−1^ (Figure 2). Upon completion of this cursory scan, the optimum conditions for chronoamperometry could be ascertained to be similar to those for the ionic liquid (at 2 V), while the current density range was greater for the acid treatments. With a composite mass of 5.85 g prior to electrochemical treatment, a mass loss of 0.045 g was calculated (*p* < 0.001; 3.405 × 10^−6^) after the post-electropolishing weight yielded a mass of 5.80 g (Table 1). 

**Figure 4:**
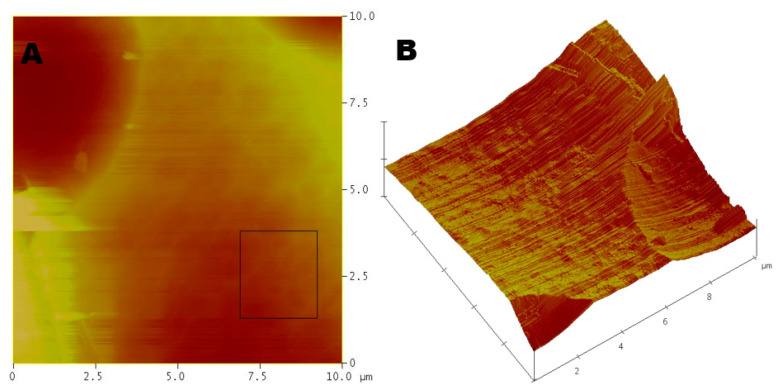
Atomic force microscopy of aluminum post-electropolishing with phosphoric acid in (**A**) 2D and (**B**) 3D—recording an average roughness of 97 ± 6 nm by utilizing the root mean square method for calculation. A 10 × 10 µm recording region was utilized.

**Figure 5:**
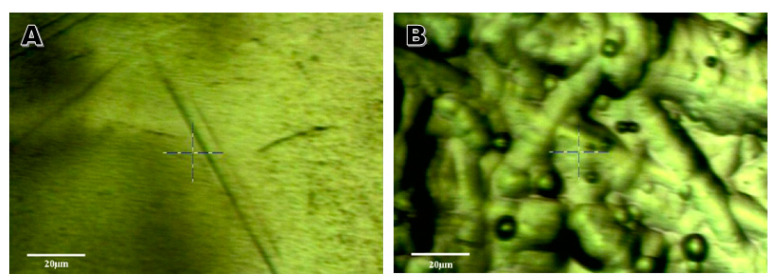
AFM console window view of aluminum samples electropolished with (**A**) an ionic liquid and (**B**) 1 M phosphoric acid, both on a 20 µm scale.

Post-treatment roughness measurements reported a root mean square roughness of 97 ± 6 nm (Figure 4), indicating that a low smoothing efficiency rating of 38.5% was achieved (Table 3). Upon comparative observation between aluminum 3D topography and AFM roughness statistics, a focused smoothness of 6 nm was achieved for a region 1/16th of the 10 × 10 µm region sampled via AFM (Figure 4). This revealed that the overall smoothness of the sample was marred by intermittent peaks and the bubbling that often occurs with pitting at low current densities during electropolishing. A representative example contrasted the two aluminum samples shown in the AFM console view, where additional evidence of this bubbling greatly affected electropolishing efficiency when surface roughness was concerned (Figure 5). Step height measurement indicated that phosphoric acid electropolishing etched an average of 8.1 µm of aluminum from the surfaces of the treated samples (Table 3).

## 3. Discussion

The phosphoric acid-facilitated aluminum polishing produced an electropolishing rate of 50.8 µg/s, which is a notably faster rate of electropolishing than the ionic liquid rate of 43.7 µg/s (Table 1). While this evidence appears favorable for the acid-polishing electrolyte, surface roughness—the primary purpose of electropolishing—was not nearly as effective for phosphoric acid, which produced an aluminum metal roughness of 97 ± 6 nm after electropolishing (Figure 4). This was calculated to an average difference of 61 nm etched from working electrode surfaces (Table 2). Step height measurements revealed that an average of 8.1 µm of aluminum material was etched away from the working electrode surface during electropolishing procedures with phosphoric acid (Table 3). When contrasted with the smaller figure of 7.2 µm returned from the ionic liquid electrolyte treatments (Table 3), it became apparent that treatments with phosphoric acid on aluminum were indeed more efficient at etching based on experiment duration and the calculated electropolishing rates from Table 1. The aluminum treatments with choline chloride and ethylene glycol had an electropolishing rate of 43.7 µg/s, whereas the phosphoric acid treatments had the superior rate of 50.8 µg/s (Table 2) [14].

This faster rate of polishing comes with the caveat of a poor surface polish in terms of overall roughness [15]. With the ionic liquid treatment of aluminum demonstrating a visibly superior roughness, with an average roughness of 26 ± 2 nm (Figure 3), when compared to the roughness of 97 ± 6 nm for phosphoric acid treatments (Figure 4), the roughness average for the acid solution has a faster polishing rate at the detriment of surface polishing efficiency (Table 2). As this smoothing efficiency is the desired design for electropolishing, the acid solution is clearly less capable than the ionic liquid mixture for creating a smooth surface (Figure 5) [14,16]. 

For the 900 s experiment duration, a chemical reaction occured at the anode immersed in the ionic liquid due to the oxidation of *Al* and the formation of *AlCl*_3_ (Equation (1)).

(1) $$Al\to 3e^{-}+Al^{3+}\;\overset{3Cl^{-}}{\to }\;AlCl_{3}$$

We observed the presence of trimethylamine, ethanol, ethylene glycol, and other products, with the incidence of trimethylamine being accounted for by Hoffman elimination of the choline base as choline hydroxide (Equation (2)):

(2) (CH3)3NCH2CH2OH+O−H→ N(CH3)3+H2O+HOCH2=CH2↔ O=CH2CH3

The reduction reaction at the cathode (counter electrode) involves the decomposition of choline by formation of a choline radical via the acceptance of an electron:

(3) (CH3)3NCH2CH2OH →+e−[(CH3)3NCH2CH2OH]▪→N(CH3)3 + C▪H2CH2OH

Thus, the transient choline radical, depicted in parentheses (Equation (3)), results from the addition of an electron from the anode at the cathode, and quickly decomposes into trimethylamine [2]. A review of the literature also indicates that the residual pitting on the surface of the metal not only affects surface reflectivity, but is also likely to affect conductivity due to increased surface area [12,17]. The plentiful abrasions and bumps generated during the electropolishing procedure (clearly visible in the AFM imagery) are a result of bubbles that formed on the metal’s surface during electropolishing. With significant proportions of the metal surface being deteriorated per second, the bubbling that occurs at the cathode can often leave these marks as they pop on the metal’s surface, sometimes marring the newly treated surface (Figure 5B) [12,18]. 

This exchange is heavily recorded in the electropolishing literature, and hydrogen evolution at the cathode (counter electrode) is a potential driver for this type of pitting. The bubbles tended to form at points in the range when low current densities occurred in the electropolishing procedure. This tended to happen towards the end of the trial, when most of the originally protruding surfaces to be polished had been removed [19]. When this occurred, the associated chronoamperogram reported a slow and steady decline, as the remaining surface area available to be electropolished slowly decreased at the rate recorded in Table 1. 

Pitting tended to occur at low current densities, or when the current applied through the 2 cm^2^ exposed surface of the metal was flowing over a decreasing surface area as the sample was being polished. This pitting is relatively inevitable, as the objective of electropolishing is to always have less surface area than prior to treatment [17]. We expect that this decreased surface area will result in an overall smoother surface. For this to occur, however, the protruding peaks must electropolish at a faster rate than the average surface upon which the roughness average calculation is based, while polishing significantly faster than any recesses in the working electrode surface. Provided that all of these interactions occur appropriately at the metal’s surface and at the proper prescribed rates, an efficient electropolishing treatment can be achieved [19].

## 4. Materials and Methods

Electrochemical tests made use of an industry standard 1 M phosphoric acid mixture for acid treatments. Acid-free treatments were carried out using an ionic liquid composed of choline chloride (Acros Organics 99%, Pittsburgh, PA, USA) and ethylene glycol (Sigma-Aldrich 99.8%, St. Louis, MO, USA); both chemicals were used as received. The ionic liquid mixture was created by stirring the two components together at a component ratio of 2 ethylene glycol: 1 choline chloride at 70 °C until a homogeneous colorless liquid emerged. This ionic liquid’s effects on electropolishing the metal surfaces of interest were analyzed using the necessary machines. Voltammetry and chronoamperometry tests were conducted using a Gamry PCI4-G750 potentiostat (Gamry Instruments, Warminster, PA, USA) and controlled using the accompanying framework and e-chem Analyst (v. 5.5) software (Gamry Instruments, Warminster, PA, USA). 

The electropolishing procedure made use of a platinum cathode (counter electrode) plate with a silver wire reference for the experimental setup. Both electrodes were degreased using deionized water and acetone to preserve the purity of samples during testing. The working electrode was abraded with 150 grit glass paper, rinsed, and dried prior to each recorded measurement to ensure reproducible voltammetric effects. Electrochemical measurements were performed at 70 °C with a constant scan rate of 20 mVs^−1^ used in voltammetric experiments. Surface analysis was carried out using a Dimension 3100 Nanoscope IV Scanning Probe Microscope, by Bruker Scientific Instruments (Billerica, MA, USA) manufactured by Digital Instruments with software version 6.12 in tapping mode. Step height measurements were recorded in µm via the Alpha Step 200 by Tencor Instruments (Milpitas, CA, USA). A KH-1300 HIROX digital microscope (Tokyo, Japan) was utilized for optical comparison to produce representative images scaled to 1600 × 1200 pixels. After completion of each experiment, weight was recorded for calculations of mass loss due to electrochemical etching.

Samples of high-purity aluminum (99.98%) were bored from supplied sheets (3 mm thickness) and labeled for use. In each test, samples of each respective metal were taped with polyimide film tape to restrict electrochemical activity to polish a 1 cm^2^ region on both the front and back faces of each metal sample. This resulted in a 2 cm^2^ region of polishing for each sample when calculating current density. Metal samples were then immersed in the electropolishing solution of choice, such that the regions for polishing were completely immersed. Aluminum samples were submitted to a cursory linear sweep voltammetry test to determine optimum voltage conditions to set for running chronoamperometry over a 900 s polishing sequence. Aluminum samples were tested with both the 1 M phosphoric acid and acid-free electropolishing solutions, upon which resulting data were managed via spreadsheet. 

## 5. Conclusions

It was determined that the phosphoric acid electrolyte mixture etched at a faster rate than the ionic liquid electropolishing treatments. This distinction is likely to be the reason that the global industry has made it and other acid solutions the standard for electropolishing. The disadvantage of this fast rate of polishing is that this acid-based method of electropolishing facilitates extensive hydrogen evolution at the working metal cathode (counter electrode), causing pitting at low current densities, or bubbling that ultimately mars the treated surfaces of acid-polished samples. The occurrence of overpotentials causes the roughness of samples to yield more favorable results in terms of average roughness with the utilization of the deep eutectic medium composed of ethylene glycol and choline chloride as compared to the acid treatment components. 

Regarding replacement of the industry standard 1 M phosphoric acid mixture, it is likely that the ionic liquid mixture could replace phosphoric acid as an efficient electrolyte for polishing, on the grounds that smoother surfaces are generated. The ionic liquid mixture provides the added benefits of recyclability without the loss of electropolishing efficiency to the nonhazardous components of which the chemical is composed. Thus, it represents an ecologically friendly supplement for the electropolishing of aluminum metals.

## Figures and Tables

**Table 1:** Average electropolishing rate (µg/s) calculations for each electrolyte for each metal sample over a 900 s treatment.

Medium	Mass Before (g)	Mass After (g)	Mass Differential (g)	Surface Degradation Rate (µg/s)
Deep Eutectic	5.83	5.79	0.039	43.77
Phosphoric Acid	5.85	5.80	0.045	50.88

**Table 2:** Roughness average (Ra) in nm for each metal post and prior to treatment with the respective solutions noted at 70 °C for 900 s. Calculated differences determined % smoothing efficiency (SE) for each sample.

Medium	Ra Before (nm)	Ra After (nm)	Ra Difference (nm)	% Ra SE *
Deep Eutectic Solvent	159.3	26.6	132.6	83.2
Phosphoric Acid	159.1	97.7	61.3	38.5

* All Smoothing Efficiency measurements reported as a %.

**Table 3:** Average recorded step heights in µm for each metal after treatment with the respective electrolytes at 70 °C for 900 s.

Medium	Step Height (µm)
Deep Eutectic Solvent	7.2
Phosphoric Acid	8.1
